# Mediating effect of symptom severity on the relationship between aggression, impulsivity and quality of life outcomes among patients with schizophrenia and related psychoses

**DOI:** 10.3389/fpsyt.2023.1154083

**Published:** 2023-09-22

**Authors:** Vathsala Sagayadevan, Pratika Satghare, Anitha Jeyagurunathan, Yen Sin Koh, Saleha Shafie, Sherilyn Chang, Ellaisha Samari, Mythily Subramaniam

**Affiliations:** Research Division, Institute of Mental Health, Singapore, Singapore

**Keywords:** aggression, mediation, impulsivity, quality of life, symptom severity

## Abstract

**Aims:**

Aggression and impulsivity among individuals with schizophrenia have been associated with poor clinical outcomes including worsening of symptoms and substance abuse which have been linked to a lower quality of life (QoL). The current study aimed to look at the mediating effect of symptom severity on the relationship between aggression, impulsivity and QoL among outpatients with schizophrenia and related psychoses in a multi-ethnic Asian population.

**Methods:**

Data (*n* = 397) were collected from outpatients seeking treatment at the Institute of Mental Health. The World Health Organization quality of life-BREF (WHOQOL-BREF) scale, the symptoms checklist-90 revised (SCL-90-R), Buss Perry aggression questionnaire (BPAQ), and the Barratt impulsiveness scales (BIS) were used to assess subjective well-being, symptom severity, aggression, and impulsivity, respectively. Mediation analysis was performed using the PROCESS macro to understand the mediating effect of symptom severity.

**Results:**

Motor impulsivity (MI) was indirectly associated with both the physical and psychological health domains of QoL while self-control was indirectly associated with the physical, psychological, and environmental health QoL domains through increased symptom severity.

**Conclusion:**

The significant indirect effect of symptom severity in our study highlights one potential pathway through which impulsivity impacts the QoL of individuals with schizophrenia and related psychoses. Elucidating other factors besides symptom severity that have an indirect effect on the QoL of individuals provides alternative approaches for treatment through which better clinical outcomes can be achieved.

## Introduction

Schizophrenia spectrum disorder is a chronic psychiatric illness affecting approximately 20 million people worldwide (1). A recent study conducted in Singapore reported that 2.3% of Singapore’s general adult population is affected by schizophrenia and related psychoses in their lifetime (2). Patients with schizophrenia spectrum disorder frequently experience deteriorated cognition that influences aggressive, impulsive tendencies that may lead to behavior problems (3, 4). Though majority of individuals with schizophrenia are not violent (5), these individuals are commonly perceived to be unpredictable, aggressive, and dangerous (6), and fears of being injured by an individual with severe mental illness are fairly common among the general public (7).

Aggression can be defined as “the intent to injure another individual using either physical or psychological means, and it has the potential to lead to violence when left unchecked (p. 897)” (8). Aggression, irritability, hostility, and anger are terms that are often used interchangeably (9). Some studies have differentiated these terms by defining anger as encompassing annoyance, hostility, and displeasure (10), irritability as sensitivity to provocation (11), and hostility as cynicism, mistrust, and denigration (9). Examining aggressive behavior among individuals is important given the personal and social costs associated with it, including physical and emotional injuries, relationship and legal problems, lower social functioning, and higher utilization of health care services (12).

Aggressive behavior is approximately 4 to 6 times higher and more commonly seen among patients diagnosed with psychiatric disorders than among the general population (13–15). A study conducted among the Swedish population found 13.2% of patients with schizophrenia to have had at least one offence of aggression as compared to 5.3% of the general population (16). Aggressive behavior among patients with schizophrenia is associated with various factors including being male (17, 18), unemployed, having a previous history of aggression (17, 19, 20), medication non-adherence, poor social support, alcohol/substance use (17, 19, 20), treatment gap (13, 21) increased stigmatization (22) and impulsivity (9, 23, 24).

Impulsivity is regarded as a multi-faceted construct (8) with a predisposition towards actions without consideration of consequences (25, 26). This includes rapid and risky decision making tendencies, poor response inhibition (27), attention deficits, and lack of planning (21). Impulsivity has been found to be positively associated with aggression [e.g., (9)]. Krakowski and Czobar (28) for instance, found baseline impulsivity (and depression) to be linked to higher levels of aggression among physically aggressive inpatients with schizophrenia. Likewise, a meta-analysis by Witt et al. (23) reported 18.5% of individuals with psychotic disorders to be violent, with impulsivity identified as a key risk factor. Despite being strongly associated with aggression; impulsivity is not routinely assessed in treatment settings (12). Studying the role of impulsivity in relation to aggression is important as it may lend explanatory power in identifying those most at risk for perpetrating aggressive behaviors (12).

In addition, despite being considered a central feature of various psychological disorders including schizophrenia (12, 26, 29, 30), the role of impulsivity among individuals with these disorders is inconclusive (26).

Quality of life (QoL) is a multidimensional construct comprising of a person’s sense of “general well-being and satisfaction with his/her life circumstances, observable social and material well-being, satisfaction with his/her social and material well-being and health and functional status” (31), p. 1226. General psychopathology such as symptoms of anxiety and depression along with positive and negative symptoms of schizophrenia have been shown to have a strong relationship to QoL. These symptoms are often disabling and pose functional threats on both personal and social fronts (31), stifling an individual’s progress in relation to work, social and life goals (32). Constructs of impulsivity and aggression have also been studied in relation to QoL. In particular, positive symptom (e.g., paranoid ideation, hostility) severity in schizophrenia has been associated with behavioral problems such as aggression [e.g., (33)], which is linked to poorer functional outcomes (31, 32, 34) including a poorer QoL (22). Aggressive tendencies in individuals, for instance, may elicit negative reactions from their surroundings, which may cause them to perceive interpersonal exchanges as being less supportive (35, 36). Furthermore, aggression levels have been linked to a poorer perception of health (37, 38). Lower satisfaction in interpersonal relationships and poorer perception of health may then result in individuals reporting lower QoL.

Likewise, Chamberlain and Grant (39) found levels of impulsivity to be responsible for variations in QoL. Non-planning impulsiveness in particular, was found to be the largest single determinant of a lower QoL among a sample of young adults in the US (39).

While some literature suggest that aggression and impulsivity may influence QoL, it is less clear how the different facets of these two constructs may affect the various QoL domains (i.e., physical health, psychological health, social relationship, and environmental). The current study aims to explore the interplay between the factors of impulsivity, aggression, symptom severity, and QoL. In particular, it seeks to examine the potential mediating effect of symptom severity on the relationship between aggression, impulsivity and QoL outcomes among outpatients with schizophrenia and related psychoses (Supplementary Figure S1).

## Methods

### Participants

Data for this study was derived from a larger study looking at aggression among patients with schizophrenia and related psychoses (40, 41). Information was collected through cross-sectional surveys conducted at the outpatient clinics at the Institute of Mental Health (IMH) between October 2019 and March 2021. The study commenced in October 2019 but was suspended from April 2020 to June 2020 due to the Coronavirus pandemic lockdown in Singapore. Adhering to safe distancing and masking policies, the study resumed in June 2020.

The study included individuals who are (i) Singapore citizens or permanent residents, (ii) aged between 21 and 65 years, (iii) able to read and understand English and (iv) have a clinical diagnosis of schizophrenia and other psychotic disorders [schizoaffective disorder, brief psychotic disorder, delusional disorder, psychosis not otherwise specified (NOS), and schizophreniform disorder] as determined by a psychiatrist based on the DSM-IV criteria. Exclusion criteria included (i) those who had intellectual disabilities or cognitive impairments, determined by clinicians prior to being referred to the study team members, (ii) were illiterate in English and (iii) were incapable of providing consent. Ethics approval was obtained from the relevant institutional ethics review board (National Healthcare Group Domain Specific Review Board) and all study procedures were conducted in accordance with the ethical guidelines. Study brochures on the ongoing study and the eligibility criteria were placed in the clinician’s rooms along with the contact details of the study team members. Treating clinicians and other health care professionals were requested to refer eligible patients to participate in the study. Interested participants provided either written or electronic informed consent (via the zoom platform) prior to data collection. Data were collected through self-administered questionnaires which participants completed either on physical copies or online via a QuestionPro link (an option provided due to the coronavirus pandemic). Of the total sample of 400 respondents, a final sample of 397 respondents was utilized for analysis. Three cases were excluded for the following reasons: being recruited twice, request for withdrawal from study, and exceeding the age limit of 65 years.

## Measures

All participants completed the following instruments as part of a study questionnaire which included:

1. Socio-demographic questionnaire: socio-demographic data collected included age, gender, ethnicity, marital status and education. All participants were asked to report information about clinical history, age of onset and clinical diagnosis. This information was also cross-checked with their medical records for all participants.
2. The World Health Organization quality of life-BREF (WHOQOL-BREF) scale: the World Health Organization quality of life-BREF (WHOQOL-BREF) scale is a 26-item scale that is a subjective evaluation of personal health and well-being over the past 2 weeks (42). The scale assesses the four domains of physical health, psychological health, social relationships, and environment and is measured using a five-point Likert scale (42). Participants were asked a range of questions including “How much you have experienced certain things in the last 2 weeks? 1 = not at all to 5 = an extreme amount/extremely; “How completely you experience or were able to do certain things in the last 2 weeks? 1 = not at all to 5 = completely; “How good or satisfied you have felt about various aspects of your life over the last 2 weeks?” 1 = very dissatisfied to 5 = very satisfied, “How often you have felt or experienced certain things in the last 2 weeks?” 1 = never to 5 = always. Questions 3, 4, and 26 were reverse-coded. The raw score for domain 1 (physical health) was obtained by summing the following questions: 3, 4, 10, 15, 16, 17, and 18. The raw score for domain 2 (psychological) was obtained by summing the following questions: 5, 6, 7, 11, 19, and 26. The raw score for domain 3 (social relationships) was obtained by summing the following questions: 20, 21, and 22. The raw score for domain 4 (environment) was obtained by summing the following questions: 8, 9, 12, 13, 14, 23, 24, and 25. The raw scores were averaged and multiplied by 4 to transform them into values between 4–20. Higher scores are reflective of greater satisfaction and higher QoL in the respective domains. The scale has been shown to have sound psychometric properties and has been validated in Singapore (43, 44).
3. The symptoms checklist-90 revised (SCL-90-R): the symptoms checklist-90 revised (SCL-90-R) is a 90-items checklist that measures psychiatric distress and severity of psychopathology symptoms (45). Respondents rated the extent to which the symptoms bothered them in the past week on a five-point Likert scale from 0 = not at all to 4 = extremely (45). Total scores for nine primary symptom dimensions and three global measures of psychological distress can be obtained from the scale. The global severity index (GSI) was calculated by taking the average of all items, with higher scores indicating greater distress and symptom severity (45). The scale has been reported to have good internal consistency (alpha coefficients 0.77–0.90), test-retest reliability, and concurrent, construct, and discriminant validity (45). While instruments such as PANSS, the SANS/SAPS, and the BPRS are typically used to evaluate symptoms in schizophrenia, the current study used the SCL-90-R. Given that the questionnaire is self-administered, the SCL-90-R, being a non-clinician administered scale was deemed suitable for the purpose of this study. While studies have highlighted potential limitations in terms of accuracy and validity of this instrument (46), it is a widely accepted screening instrument (47) which has been used in a variety of clinical settings (46) among various populations including Schizophrenia populations (48–50) and First Episode Psychosis (FEP) populations [e.g., (51)].
4. Buss Perry aggression questionnaire (BPAQ): the Buss Perry aggression questionnaire (BPAQ) is a 29-item, four-factor instrument (52) measuring physical aggression, verbal aggression, anger, and hostility. Items are rated on a five-point Likert rating scale, where 1 = extremely uncharacteristic of me, 2 = somewhat uncharacteristic of me, 3 = neither uncharacteristic nor characteristic of me, 4 = somewhat characteristic of me, and 5 = extremely characteristic of me. The total BPAQ score ranges from 29 to 145 with higher scores indicative of higher levels of aggression. Subscale scores were obtained by summing the ratings for the questions that define each of the subscales. The scale has been shown to have high internal consistency and a valid factor structure among outpatients with schizophrenia and related psychoses (40).
5. Barratt’s impulsivity scale-11 (BIS-11): the Barratt’s impulsivity scale-11 (BIS-11) is a 30-item self-report questionnaire assessing the multi-faceted personality/behavioral construct of impulsiveness (53). It is rated on a four-point Likert scale from 1 = rarely/never to 4 = almost always/always. The total scores range from 30 to 120. Higher scores on the BIS reflect higher levels of impulsiveness (53). Based upon Lau’s et al. (41) paper which validated the BIS-11 scale with the same study sample, a three-factor model was used: non-planning impulsivity (items 1, 7, 12, 13, and 20, all reverse-coded), motor impulsiveness (items 2, 11, 14, 17, 18, and 19), and lack of self-control (items 22, 24, 25, 26, and 27). Examples of items from each subscale are “I act on the spur of the moment” (motor impulsiveness), “I change hobbies” (self-control), and “I plan tasks carefully” (non-planning impulsiveness). The BIS-11 has shown good internal consistency in various populations, with Cronbach’s alpha ranging from 0.71 to 0.83 (54).

## Statistical analysis

IBM SPSS Statistics for Windows, version 23 was used for all analyses, with two-sided tests at a significance level of 5%. Mean and standard deviation (SD) were presented for continuous variables, whereas count and percentages were presented for categorical variables. Pearson’s correlation was used to examine the association between the variables of interest. Mediation analysis was performed using PROCESS macro (55). Results were presented in beta-coefficients and 95% confidence intervals. Bias-corrected bootstrapping with 5,000 bootstrap samples was conducted to obtain the 95% confidence interval (CI). The indirect effect is considered to be significant if the 95% CI does not cross 0. The mediation analysis was controlled for the following covariates: age, gender, ethnicity, education, and marital status. As the assumption of homoscedasticity was violated, robust variance estimation (HC4 estimator) was included in the mediation analysis. Bonferroni correction was done to correct for test multiplicity.

## Results

### Sample characteristics

The mean age of the sample was 36.2 years (SD = 10.9). The majority of the sample was composed of males (50.6%), those of Chinese ethnicity (74.8%), having A-level/polytechnic/vocational School/ITE education (45.8%), and were single (80.6%) (Table 1). Mean scores of the BIS, BPAQ, SCI-90-R and the WHOQOL-BREF scale are also reflected in Table 1.

**Table 1:** Sample characteristics and mean (SD) scores of scales.

Variables	*n* (%)
*Gender*	
Male	201 (50.6)
Female	196 (49.4)
*Ethnicity*	
Chinese	297 (74.8)
Malay	51 (12.8)
Indian	38 (9.6)
Others	11 (2.8)
*Education*	
Secondary and below	138 (34.8)
A-Level/polytechnic/vocational school/ITE^*^	182 (45.8)
Degree and above	77 (19.4)
*Marital status*	
Single	320 (80.6)
Married	47 (11.8)
Separated/divorced/widowed	30 (7.6)

	Mean (SD^*^)
Age	36.2 (10.9)
*Barratt impulsiveness scale (BIS)*	
BIS-non-planning impulsivity score^a^	12.0 (3.5)
BIS-motor impulsivity score^a^	11.5 (3.9)
BIS-lack of self-control score^a^	9.9 (3.3)
Symptoms checklist-90 revised (SCI-90-R) global severity index score^a^	0.9 (0.9)
*Buss–Perry aggression questionnaire (BPAQ)*	
BPAQ-anger score^a^	14.3 (5.5)
BPAQ-physical aggression score^a^	19.7 (7.6)
BPAQ-verbal aggression score^a^	9.9 (3.7)
BPAQ-hostility score^a^	21.6 (7.4)
*The World Health Organization quality of life-BREF (WHOQOL-BREF) scale*	
QOL-physical health score	14.1 (2.9)
QOL-psychological health score	13.3 (3.2)
QOL-social relationships score	12.9 (3.6)
QOL-environment score	14.0 (3.0)

^*^SD, (standard deviation); ^*^ITE, (Institute of Technical Education).

a Missing data: BIS-non-planning impulsivity (*n* = 3), BIS-motor impulsivity (*n* = 11), BIS-lack of self-control (*n* = 7), symptom severity (*n* = 29), BPAQ-anger (*n* = 1), BPAQ-physical aggression (*n* = 1), BPAQ-verbal aggression (*n* = 1), BPAQ-hostility (*n* = 5).

### Correlation between impulsivity, aggression, symptom severity and QoL

All 3 factors of impulsivity: non-planning impulsivity, MI, and lack of self-control and all four factors of aggression: Anger, physical aggression, verbal aggression, and hostility were negatively associated with all four domains of QoL and positively correlated with symptom severity. Aggression was also positively associated with impulsivity with most correlations being significant at *p* = 0.01. The correlations between the various variables are presented in Table 2.

**Table 2:** Correlation between impulsivity, aggression, symptom severity and QoL.

	BIS-non-planning impulsivity	BIS-motor impulsivity	BIS-lack of self-control	BPAQ-anger	BPAQ-physical aggression	BPAQ-verbal aggression	BPAQ-hostility	Physical health	Psychological	Social relationships	Environment	SCI-90-R global severity index
BIS-non-planning impulsivity		0.20^**^	0.03	0.21^**^	0.17^**^	0.11^*^	0.12^*^	−0.29^**^	−0.33^**^	−0.22^**^	−0.37^**^	0.15^**^
BIS-motor impulsivity			0.57^**^	0.39^**^	0.35^**^	0.36^**^	0.40^**^	−0.38^**^	−0.38^**^	−0.25^**^	−0.27^**^	0.55^**^
BIS-lack of self-control				0.34^**^	0.33^**^	0.37^**^	0.40^**^	−0.30^**^	−0.26^**^	−0.29^**^	−0.23^**^	0.54^**^
BPAQ-anger					0.77^**^	0.74^**^	0.71^**^	−0.28^**^	−0.24^**^	−0.27^**^	−0.27^**^	0.43^**^
BPAQ-physical aggression						0.71^**^	0.71^**^	−0.27^**^	−0.23^**^	−0.27^**^	−0.29^**^	0.38^**^
BPAQ-verbal aggression							0.72^**^	−0.24^**^	−0.20^**^	−0.22^**^	−0.22^**^	0.43^**^
BPAQ-hostility								−0.31^**^	−0.34^**^	−0.37^**^	−0.29^**^	0.50^**^
Physical Health									0.72^**^	0.60^**^	0.69^**^	−0.60^**^
Psychological										0.62^**^	0.69^**^	−0.58^**^
Social relationships											0.60^**^	−0.47^**^
Environment												−0.45^**^
SCI-90-R global severity index												

^**^Correlation is significant at the 0.01 level (2-tailed). ^*^Correlation is significant at the 0.05 level (2-tailed).

### Mediating effect of symptom severity on the association between impulsivity, aggression and QoL

Table 3 presents the potential mediating effect of symptom severity on the relationship between aggression, impulsivity and QoL outcomes among outpatients with schizophrenia and related psychoses.

**Table 3:** Indirect effect of symptom severity on the association between impulsivity, aggression and QoL.

	Total effects		Direct effects		Indirect effects		
	*β*	SE	*β*	SE	*β*	SE	95% CI
*Outcome: physical health*							
BIS-non-planning impulsivity	−0.18^*^	0.06	−0.16^*^	0.05	−0.02	0.03	−0.07 to 0.03
BIS-motor impulsivity	−0.17	0.06	−0.06	0.05	−0.11^*^	0.03	−0.18 to −0.05
BIS-lack of self-control	−0.08	0.07	0.04	0.06	−0.13^*^	0.03	−0.19 to −0.07
BPAQ-anger	0.01	0.05	0.04	0.04	−0.03	0.02	−0.07 to 0.02
BPAQ-physical aggression	−0.003	0.04	−0.02	0.03	0.02	0.02	−0.01 to 0.05
BPAQ-verbal aggression	0.03	0.08	0.06	0.06	−0.03	0.03	−0.09 to 0.04
BPAQ-hostility	−0.06	0.04	−0.007	0.03	−0.05	0.02	−0.08 to −0.02
*Outcome: psychological*							
BIS-non-planning impulsivity	−0.22^*^	0.06	−0.21^*^	0.05	−0.02	0.03	−0.08 to 0.03
BIS-motor impulsivity	−0.15	0.07	−0.03	0.05	−0.12^*^	0.03	−0.19 to −0.06
BIS-lack of self-control	−0.08	0.07	0.06	0.06	−0.14^*^	0.04	−0.21 to −0.07
BPAQ-anger	0.04	0.06	0.07	0.05	−0.03	0.03	−0.08 to 0.03
BPAQ-physical aggression	0.02	0.04	0.002	0.03	0.02	0.02	−0.01 to 0.06
BPAQ-verbal aggression	0.08	0.08	0.10	0.07	−0.03	0.04	−0.10 to 0.04
BPAQ-hostility	−0.15^*^	0.04	−0.09	0.03	−0.06	0.02	−0.09 to −0.02
*Outcome: social relationship*							
BIS-non-planning impulsivity	−0.18	0.08	−0.16	0.07	−0.02	0.02	−0.06 to 0.03
BIS-motor impulsivity	−0.01	0.08	0.08	0.07	−0.10	0.03	−0.16 to −0.03
BIS-lack of self-control	−0.19	0.09	−0.08	0.09	−0.11	0.03	−0.17 to −0.05
BPAQ-anger	−0.008	0.06	0.01	0.06	−0.02	0.02	−0.06 to 0.02
BPAQ-physical aggression	−0.006	0.04	−0.02	0.04	0.01	0.01	−0.01 to 0.04
BPAQ-verbal aggression	0.12	0.09	0.15	0.08	−0.02	0.03	−0.08 to 0.03
BPAQ-hostility	−0.14	0.05	−0.10	0.04	−0.04	0.02	−0.08 to −0.01
*Outcome: environment*							
BIS-non-planning impulsivity	−0.25^*^	0.06	−0.23^*^	0.06	−0.01	0.02	−0.06 to 0.02
BIS-motor impulsivity	−0.04	0.06	0.04	0.05	−0.08	0.03	−0.14 to −0.03
BIS-lack of self-control	−0.10	0.08	−0.01	0.07	−0.10^*^	0.03	−0.15 to −0.05
BPAQ-anger	0.02	0.05	0.04	0.05	−0.02	0.02	−0.05 to 0.02
BPAQ-physical aggression	−0.03	0.04	−0.05	0.03	0.01	0.01	−0.009 to 0.04
BPAQ-verbal aggression	0.05	0.08	0.07	0.07	−0.02	0.02	−0.07 to 0.03
BPAQ-hostility	−0.06	0.04	−0.02	0.04	−0.04	0.01	−0.06 to −0.01

^*^p-value <0.05, corrected for multiple testing.

#### Physical health QoL domain

Higher MI (*β* = −0.11, 95% CI: −0.18 to −0.05), and higher lack of self-control (*β* = −0.13, 95% CI: −0.19 to −0.07) were indirectly associated with poorer physical health through symptom severity. After accounting for this mechanism, there was no direct effect between these factors and physical health.

#### Psychological health QoL domain

Higher MI (*β* = −0.12, 95% CI: −0.19 to −0.06), and higher lack of self-control (*β* = −0.14, 95% CI: −0.21 to −0.07) were indirectly associated with poorer psychological health through symptom severity. After accounting for this mechanism, there was no evidence of direct effect between these factors and psychological health.

#### Environmental QoL domain

Only higher lack of self-control (*β* = −0.10, 95% CI: −0.15 to −0.05), was indirectly associated with lower environmental *QoL* scores through symptom severity. After accounting for this mechanism, there was no evidence of direct effect between this factor and the environmental *QoL* scores.

## Discussion

Overall, self-control and MI were indirectly associated with lower QoL across the physical and psychological health QoL domains. Only self-control was indirectly associated with lower environment health QoL scores. No associations were found for both impulsivity and aggression with the social relationships QoL domain. The first section will discuss the indirect effect of lack of self-control on the physical, psychological, and environmental QoL health domains. The next section will discuss the indirect effect of MI on both the physical and psychological health QoL domains.

### The indirect effect of lack of self-control on QoL

#### Physical health domain

Lack of self-control and its detrimental effect on physical health has received considerable support. The tendency towards impulsivity and inability to delay gratification among these individuals places them at a higher risk of developing serious health conditions (56). Moffitt et al. (57) for instance, followed children from birth through the age of 32 years to examine how childhood self-control is predictive of outcomes such as physical health and substance dependence. Childhood self-control was found to be predictive of adult health problems (e.g., cardiovascular, respiratory, dental, and sexual health, inflammatory status) even after accounting for social status and IQ (57). A similar finding was reported by Miller et al. (56) who explored the relationship between different levels of self-control during adolescence and the likelihood of developing various physical and brain-based health conditions in adulthood. Those with lower self-control had higher odds of being diagnosed with physical conditions such as asthma, cancer, high cholesterol, and high blood pressure and brain-based conditions such as depression, and attention deficient hyperactivity disorder (ADHD) (56).

While the aforementioned studies offer support for the direct effect of a lack of self-control on physical health, the indirect effect of self-control on physical health can be partially accounted for by the self-control theory (56). In accordance with the self-control theory, low levels of self-control lead to health-risk behaviors such as smoking or substance abuse (57) which may influence the severity of symptoms. Furthermore, those with lower levels of self-control are more likely to place themselves in situations that pose a risk of injury or death (56). Wills et al. (58) for instance, found high self-control to be linked to greater fruit and vegetable intake, more involvement in sports and lesser sedentary behavior among a multi-ethnic sample of school students. In contrast, poorer self-control was associated with greater saturated fat intake and less vigorous exercise (58). As such, the tendency for individuals with low self-control to place themselves in situations that pose considerable risks and engage in behaviors that may potentially aggravate their symptoms may have contributed to a poorer QoL within the physical health domain.

#### Psychological health domain

Several studies which have focused on self-control have examined this construct among the younger population, given that levels of self-control developed in the younger years are often predictive of long-term outcomes such as physical and mental health, education levels, career opportunities, and financial security (4, 59).

There is evidence to suggest that mental health problems are related to structural and functional abnormalities in the prefrontal cortex which may impair executive functions (3, 4) such as inhibition. The current study sample consisted of individuals with schizophrenia and related psychoses; thus, impaired executive functions may have resulted in lower levels of self-control reported among this population. Furthermore, the association between self-control and mental health is thought to be reciprocal, such that lower levels of self-control reported among these individuals may affect their sense of self-efficacy related to work and daily activities of living which may worsen symptoms of anxiety or depression (4, 60). Hence, lower levels of self-control may influence symptom severity through factors such as self-efficacy which in turn affect the mental health of individuals. While the current study found self-control to affect psychological health through symptom severity, there are likely other factors that moderate the link between self-control and symptom severity itself.

#### Environmental health domain

The environmental domain of QoL in the current study was assessed through items measuring the feeling of safety, satisfaction to access to health services, living conditions, information, opportunity for leisure activities, as well as the ability to finance one’s own needs. Compared to the other domains of QoL, it is less clear how lack of self-control might indirectly affect the environmental domain through symptom severity. The effect of MI and lack of self-control on the other QoL domains may in some way influence this domain. The extent to which one is able to meet their needs, be satisfied with health services or transportation or have access to relevant information to function in their daily life is likely dependent on their physical and psychological health. These factors may serve to hinder or facilitate the ability of an individual to navigate their environment and fulfil their daily activities. For instance, impulsive behavior such as lack of self-control and MI may reduce one’s ability to pursue long-term goals and cater to one’s own needs (61). This then, creates an environment that is relatively unstable and unconducive for an individual to thrive in.

### The indirect effect of MI on QoL

#### Physical health domain

Similar to lack of self-control, MI was found to be indirectly associated with the physical health QoL domain through symptom severity. MI might manifest as the inability to control, stop or cancel motor patterns as often seen in individuals with ADHD (62, 63).

Some studies have found substance use to be associated with MI. Wagner et al. (64) for instance, found MI to be significantly associated with alcohol use among a sample of individuals with cannabis use disorder, with greater alcohol use being associated with greater difficulties in being able to inhibit their responses (64). Similarly, Fox et al. (65) found MI (among other factors) to be independently predictive of alcohol use among a community sample of regular drinkers. Chang et al. (66) who utilized the same study sample as in the current paper found the prevalence of problematic drug use and/or problematic alcohol use to be 10.6% in this sample. Among these individuals, those with greater symptom severity were also twice as likely to have problematic drug use and/or alcohol use (66).

Given the presence of co-morbid substance use disorders in this sample, co-morbid alcohol use may not only worsen clinical outcomes (i.e., more severe psychotic and depressive symptoms) (65, 67) but may as a consequence, negatively impact an individual’s physical well-being and QoL. In a similar vein, constant physical and emotional agitation may result in disturbed sleep that worsens symptoms in turn, interfering with daily functioning, mood, energy levels, and memory (68). However, it is important to note that psychiatric disorders are often related to sleep problems that may also increase the risk of impulsivity (68).

#### Psychological health domain

Higher MI was also found to be indirectly associated with poor psychological health through symptom severity in the current study. MI in the present study was measured through items assessing restlessness, awkwardness, poor concentration, as well as problematic behavior involving the tendency to act on a whim without any forethought (53).

While not specific to MI, there is some evidence to suggest an association between impulsivity and general psychopathology, namely, positive and negative symptoms of schizophrenia (69, 70). Impairments in motor processing and control are characteristic of schizophrenia, with motor dysfunctions having been linked to memory, executive functioning, and attention deficits (26, 71). It is likely that motor abnormalities and related deficits in higher-level cognitive functions cause individuals to exhibit rapid, abrupt reactions to external or internal stimuli, and thoughts without gauging the consequences of their reactions for themselves or others (25). A population-based study (72) and a study conducted among psychiatric outpatients diagnosed with schizophrenia (41) for instance, found more severe psychological symptoms to correlate with greater impulsivity.

Although in the current study, MI was found to influence psychological health through symptom severity, it is likely that patients with greater symptom severity are more likely to experience poor inhibitory control, neurocognitive impairments and thus, higher dysfunctional impulsive behavior (73). Heightened parietal cortex activity (74) associated with the presence of high levels of positive and negative symptoms, has been shown to account for impaired motor inhibitory control and greater MI (75). Paulik et al. (76), suggested a possible link between the presence of increased frequency of hallucinations and impaired intentional control of intrusive cognition. This impairment may then lead to a lack of insight, and memory deficit which can potentially aggravate negative symptoms of apathy, anhedonia, anxiety, and depression among patients with schizophrenia leading to poor psychological QoL (22).

Interestingly, aggression was not found to be directly or indirectly associated with QoL in the current study. Previous studies have shown some aspects of aggression such as hostility and anger to be associated with poorer general health perceptions (37), quality of social interactions (35, 38), poorer mental health of individuals (77) as well as lower QoL (78). While we are unable to account for this anomalous result, one reason for its non-significance might be related to medication non-adherence (79), given that atypical antipsychotic medications have been shown to ameliorate hostility and aggressive behavior associated with psychosis (80). For example, Alia-Klein et al. (81) who studied psychotic inpatients, detained at a forensic unit in New York, found the majority of individuals who were medication non-adherent to have engaged in physical assault as compared to only one-third of the adherent group. Given that our sample consisted of outpatients who are relatively stable, it is possible that medication non-adherence and aggressive tendencies might be much lower in this population. However, this is speculative as medication non-adherence was not examined in this sample.

## Limitations

In considering the results of the current study, it is important to note some of the limitations present. Firstly, the cross-sectional nature of the study limits the ability to identify causal relationships between aggression, impulsivity, symptom severity and QoL. Secondly, the current study did not measure the presence of comorbid disorders and the total number of psychiatric diagnoses. While a previous paper using the same study sample established the prevalence of problematic drug/alcohol use in this population (66), the presence of other co-morbid disorders were not identified. Greater ADHD symptom severity, for example, has been associated with increased aggression (82) and impulsivity (83). Not taking into consideration the presence of co-morbid disorders in the current sample could be a potential confounding factor given that those with several co-morbid disorders would likely report greater symptom severity as well as poorer QoL.

Terms such as QoL have also been defined differently across studies and have been assessed using various scales. While the current study used the World Health Organization Quality of Life-BREF (WHOQOL-BREF) scale encompassing the physical health, psychological health, environment and social relationships domains, other studies have utilized scales such as SF-36, a self-report measure of physical and mental Health-related Quality of Life (HRQoL) which covers eight primary dimensions of subjective health perception including “physical functioning, role limitations due to physical problems, bodily pain, general health perceptions, vitality, social functioning, role limitations due to emotional problems and mental health.” [(84); p. 127 (85)]. It is thus important to take this into consideration given that defining and assessing the constructs in different ways will likely influence the results of studies.

One other limitation is the presence of similar items on the BIS/BPAQ and the SCL-90-R. For instance, both the physical aggression subscale of the BPAQ and the hostile subscale of SCL-90-R have similar items measuring the tendency to break things: “I have become so mad that I have broken things” (physical aggression subscale-BPAQ) and “Having urges to break or smash things” (hostile subscale-SCL-90-R). This might have contributed to an artefactual relationship between impulsivity, aggression and the SCL-90-R global score.

Lastly, urgency, a construct of impulsivity, and its underlying neural circuitry are seen to be responsible for aggressive behavior and heightened emotions (18). Experiencing significant heightened emotions poses substantial risk for violence, substance use and suicidality (18). Among patients with schizophrenia, urgency may have contributed to aggressive behavior and may mediate this relationship. The use of Barratt Impulsivity scale in the current study did not comprise of this construct which could potentially limit the understanding of the role of urgency in relation to impulsivity and aggression.

## Conclusion

Although the current study explored the uni-directional influence of aggression and impulsivity on symptom severity and QoL, the links between these factors are likely bi-directional. Nevertheless, the significant indirect effect of symptom severity highlights one potential pathway through which impulsivity impacts the QoL of individuals with schizophrenia and related psychoses. Individuals who display high levels of impulsivity may benefit from additional support such as the development of specific skills to improve their functioning in the community including behavioral interventions and communication skills training (12). Future studies can explore other factors besides symptom severity that have an indirect effect on the QoL of individuals. This may facilitate alternative approaches for treatment as well as adaptation of current therapeutic strategies to ameliorate the adverse effects of impulsive behavior on QoL outcomes.

## Data availability statement

Data may be available upon reasonable request and subjected to approval by the institutional review board (IRB). This is a requirement mandated for this research study by our IRB and funders. Requests to access the dataset should be directed to the senior author, MS, mythily@imh.com.sg.

## Ethics statement

The studies involving humans were approved by National Healthcare Group Domain Specific Review Board. The studies were conducted in accordance with the local legislation and institutional requirements. The participants provided their written informed consent to participate in this study.

## Author contributions

VS and PS wrote the manuscript. AJ conceived the study and wrote the protocol. YSK analysed the data and assisted in the interpretation of the findings. SS, SC, and ES provided comments for the article. MS reviewed the protocol and critically reviewed the article. All authors contributed to the article and approved the submitted version.
