# Supplementary material for: Mediating effect of symptom severity on the relationship between aggression, impulsivity and quality of life outcomes among patients with schizophrenia and related psychoses
Source: Front Psychiatry. 2023 Sep 22;14:1154083. doi: 10.3389/fpsyt.2023.1154083 (PMC10556254; doi:10.3389/fpsyt.2023.1154083)
Supplement: Supplementary file 1 [file Data_Sheet_1.pdf]

Supplementary Material

Mediating effect of symptom severity on the relationship between Aggression, Impulsivity and Quality of Life outcomes among patients with Schizophrenia and related psychoses

Vathsala Sagayadevan*[1†], Pratika Satghare[1†], Anitha Jeyagurunathan[1], Yen Sin Koh[1], Saleha Shafie[1], Sherilyn Chang[1], Ellaisha Samari[1] and Mythily Subramaniam[1]

[1] Research Division, Institute of Mental Health, Singapore, Singapore

[†]These authors contributed equally to this work and share first authorship

***Correspondence:**

Vathsala Sagayadevan: Vathsala_SAGAYADEVAN1@imh.com.sg

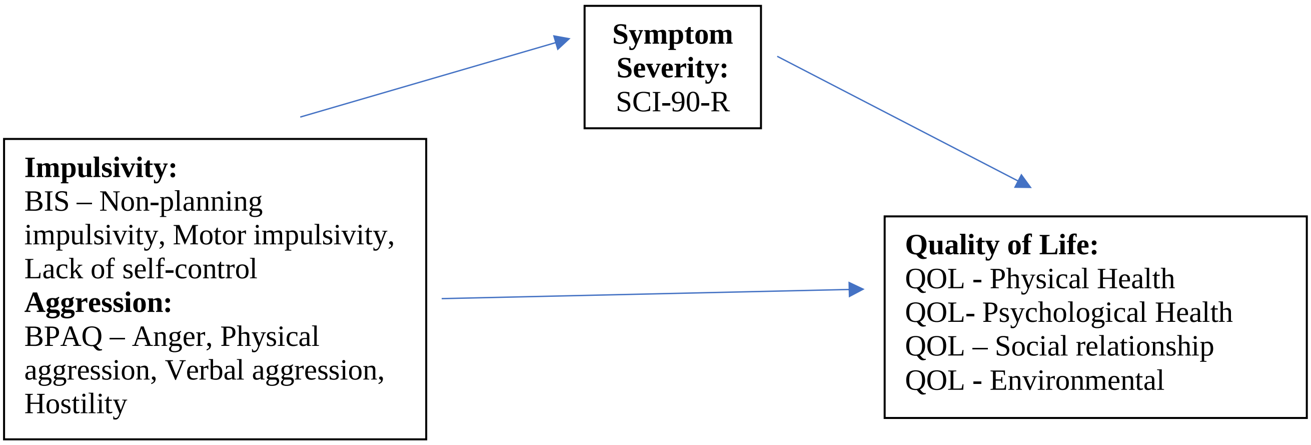

Supplementary Figure 1.

Theoretical Mediation Model

Legend:

Symptom Severity: Symptoms Checklist-90 Revised (SCI-90-R)
Impulsivity: Barratt's Impulsivity Scale-11 (BIS)
Aggression: Buss Perry Aggression Questionnaire (BPAQ)
Quality of Life (QOL): The World Health Organization Quality of Life-BREF (WHOQOL-BREF) scale
